# Supplementary material for: Are carbon emissions trading and green financial instruments synergistic? -Comprehensive quantitative research based on content analysis
Source: PLoS One. 2024 Mar 7;19(3):e0298601. doi: 10.1371/journal.pone.0298601 (PMC10919601; doi:10.1371/journal.pone.0298601)
Supplement: S1 Data — (ZIP) [file pone.0298601.s001.zip › Data/地级市Z_F工作报告.zip!/╜¡╬≈/╥╦┤║╩╨/└┤╫╘╣1⁄2╓┌║┼í╛┬φ┐╦╩2╛▌═°í┐_2013.docx]

各位代表：

　　现在，我代表市人民政府向大会报告工作，请予审议，并请市政协委员和列席会议的同志提出意见。

一、2012年工作回顾

　　刚刚过去的2012年，是本届政府全面履职的起始之年。一年来，全市人民在省委、省政府和市委的正确领导下，深入贯彻科学发展观，加速推进幸福宜春建设，迎难而上，奋力拼搏，在困境中寻发展、在挑战中促提升，完成了市三届人大二次会议确定的各项目标任务。

　　（一）赶超发展有新突破。预计全年完成生产总值1250亿元，增长11%；财政总收入实际完成200.4亿元，增长34.3%，其中地方财政收入132.4亿元，增长43.4%；500万元以上固定资产投资1020亿元，增长30.8%；规模以上工业增加值500亿元，增长15.7%，新增规模以上工业企业72户，净增64户。县域经济实力增强，财政总收入超过10亿元县市区达到8个，丰城蝉联“县域经济百强县市”，居全国第93位，比上年前移4位，樟树被评为“全省县域经济发展十大活力县市”并荣登榜首。进位赶超取得新成果，经济发展实现“一突破、四跨越、七靠前”：工业园区主营业务收入突破两千亿元；财政总收入、地方财政收入、规模以上工业增加值、固定资产投资分别跨越两百亿、百亿、五百亿、千亿大关；财政总收入和地方财政收入增速、工业用电总量、中石化成品油销量增幅、外贸出口增幅、新增贷款及增幅均居全省前列。

　　（二）项目建设有新成效。以“主攻重大项目、推进产业升级”活动为抓手，打响项目建设“夏季大会战”，开展“三千干部进企业、助推决战五百亿”帮扶活动，坚持重大项目协调推进机制，适时到县市区和部门召开现场办公会，解决项目用地、资金等难题240多个，促进了一大批项目快签约、快落地、快投产。全年引进省外5000万元以上项目170个，项目个数连续五年居全省第一，其中，引进10亿元以上项目48个；成功包装项目118个，争取省级用地指标 3.5万亩；新开工亿元以上项目195个，10亿元以上项目40个。锂电新能源产业进一步壮大，共签约项目88个，其中进资、动工或投产企业62家，实际进资59.4亿元。河北御捷、赣锋锂业、格林美、中国瑞林、广州王老吉、金达莱环保、紫宸科技等项目相继落户；宜春重工、明冠能源、中天机械、宏宇能源、科伦医疗器械、新威动力等项目投产见效；明月山机场、明月大道、文化之窗、花博园、奉铜高速等项目全面完工；明月大桥、宜春新火车站综合交通枢纽、靖安洪屏抽水蓄能电站、杭长铁路客运专线、万宜高速公路、昌樟高速公路改扩建、丰厚一级公路等项目加快推进。高安八景至重庆团结村集装箱“五定班列”顺利开通。宜春经开区申报国家级开发区取得实质性进展。奉新工业园区被命名为“中国纺织产业转移试点园区”。

　　（三）城乡面貌有新变化。按照 “三高”要求，突出抓好“四大一重”，城建三年大会战圆满收官，全市在建项目221个，竣工项目416个，累计完成投资466亿元。城镇化率达44.7%，比上年提高2个百分点。环城南路顺利开工，全市城市建成区面积248平方公里，其中中心城区60平方公里。成功入选中国“十佳宜居城市”，并荣获“最具幸福感城市”。樟树、万载被命名为“省级园林城市”，铜鼓被命名为“省级生态县”，丰城荣获“全国城市管理进步奖”。中心城区完成50条小街小巷改造，启动18座开放式运动游园建设，市容环境暗访连续七年居全省第一。温汤镇“六位一体”改造全面完工，成为全省示范镇样板。新农村建设迈入全省先进行列。全市村镇建设投入35亿元。实施高速公路沿线44个乡镇秀美乡村提升工程。丰城在全省率先实现农村公路“建管养运”一体化全覆盖。靖安在全省率先启动城乡一体化垃圾处理工程。全市粮食播种面积930.8万亩，总产400.4万吨，实现粮食生产“九连丰”，收购总量居全省第一，荣获“全国粮食生产先进市”，丰城、高安、樟树再次获得“全国粮食生产先进县市”。农业产业化步伐加快，农业产业化龙头企业增至222家，其中国家级6家、省级65家。农民专业合作社突破2000家，其中省级示范社65家。万载获批“国家级现代农业示范区”。袁州等6县市区被列为“国家油茶产业发展重点示范基地”。宜丰基层农技推广服务体系改革与建设经验在全省推广，靖安、奉新、宜丰和万载被列为“国家示范县”。宜丰荣获“中国林业产业兴林富民示范县”。奉新被授予“中国竹产业示范县”。丰城富硒有机稻标准示范区荣获“全国农业标准化示范区”，高安被定为“全国无公害蔬菜生产基地”，靖安白茶获批国家地理标志保护产品。全市集中清理处置闲置工业用地1万余亩，新增耕地4.1万亩，我市荣获全省首个“全国国土绿化突出贡献单位”，上高获得“全国首届国土资源节约集约模范县”。强化“既不能守着青山绿水受穷，也不能踩着污水垃圾致富”的理念，深入开展六大环保专项整治，生态环境有效改善。39座大中型水库、838座小型水库退出承包养殖，高安荣获“全国农田水利基本建设先进县市”，袁州河道采砂整治做法在全省推广。

　　（四）民生改善有新提升。大力实施“十大幸福工程”，加大民生投入，各类民生支出占财政总支出的70%以上。预计城镇居民人均可支配收入18896元，增长15%；农民人均纯收入7993元，增长14.5%。市场物价基本稳定，预计社会消费品零售总额350亿元，增长13%。新增城镇就业6.5万人，转移农村富余劳动力 11.2万人，就业工作连续10年列全省第一，荣获“全国创业先进城市”，市就业局被评为“全国就业工作先进单位”。社会保障体系更加完善，保障水平大幅提高，丰城城乡大病医疗救助标准和封顶线提升到全省最高水平。民政工作稳步推进，中心城区最低生活保障标准提高至每人每月370元，列全省第二，市民政局荣获“全国民政系统先进集体”，万载荣获“全国村务公开民主管理示范县”和“全国双拥模范县”，万载农保局获“全国新型农村和城镇居民养老保险先进单位”，袁州民政局婚姻登记处被评为“全国民政系统群众满意窗口”。建成保障性住房12414套、棚户区改造9247套、农村危房改造9100户。完成农村饮水安全工程39处，解决40.5万农村居民饮水安全问题。“万村千乡市场工程”不断提升，完成农家店信息化改造730家，列全省第二。实施35所城区中小学新建、改扩建工程，新增学位5.4万个，其中中心城区新建、改扩建学校12所，新增学位1.6万个，特别是新建宜春特教学校，为残疾少年儿童就读创造了良好条件。我市被国务院评为全国“两基”工作先进地区，上高荣获“全省教育工作目标考核先进县”。宜春职业技术学院跻身省级示范性高职院校行列，宜春一中恢复独立办学。中心城顺利通过国家卫生城市复审，再次荣膺“国家卫生城市”称号。宜春市中医院被评为全市首家“国家三级甲等中医院”。全民健身工作走在全省前列。成功举办第六届月亮文化节、省第三届花博会等大型活动。所有行政村设有农家书屋。广播“村村响”任务全面完成。认真落实人口计生政策，出生人口性别比趋于正常。上高荣获“全国阳光计生行动示范单位”，铜鼓、高安分别被授予“中国长寿之乡”、“中国书法之乡”。全市荣登2012年中国好人榜29人，居全省第一。旅游工作成效显著，预计接待游客1685万人次，增长46.8%；实现旅游综合收入124亿元，增长43.9%。明月山景区跃居全省四大景区之一，门票收入突破亿元。央视“北纬30度中国行”、“城市1对1”、“寻宝”等栏目走进宜春，宜春的知名度和美誉度进一步提升。深入开展专项整治，有效防范和遏制重特大安全事故，安全生产态势平稳好转。加强应急体系、预警机制和救援队伍建设，突发事件处置能力有效提高。开展万名干部“三进四民”、千名干部“安全稳定大督查”等活动，突出隐患排查，做好信访积案化解，帮助解决了一大批群众生产生活难题，受到普遍称赞。推进食品药品安全示范市创建，完善社会治安防控体系和“天网工程”，严厉打击各类违法犯罪，群众安全感不断增强。

　　（五）改革开放有新拓展。国有集体企业改革扫尾工作有序推进，全市纳入本轮改革的107户七个系统企业改革基本完成，被评为全省先进市。医药卫生体制改革扎实推进，全市183家乡镇卫生院、21家社区卫生服务中心、2460个村卫生室实施国家基本药物制度。国库集中收付改革深入实施，政府采购改革完成管采分离。文化体制改革加快推进，完成市采茶歌舞剧院等8个国有艺术院团转企改制。开放型经济迅速发展，预计全年实际利用外资4.8亿美元，增长11.4%；外贸出口13.7亿美元，增长36.5%；高安国家建筑卫生陶瓷监督检测中心获批建设，宜春海关设立工作有序推进，高安铁路口岸作业区、万载公路口岸作业区建设进展顺利。民营经济进一步壮大，预计实现营业收入2800亿元，增长30%；上缴税金120亿元，占全市税收收入的80%以上。金融支持地方经济力度加大，全市信贷净增额突破140亿元，增长62.8%，增幅居全省第一；保费收入总量居全省前列，财产险增幅列全省第一。交通银行、兴业银行相继落户，宜春农商行挂牌营业，丰城成功发行全省首单“中小企业集合票据”。企业上市工作取得新突破，宜丰万国矿业成功在香港上市，袁州英龙橡胶在天津股权交易所上市交易，成为我省首批在“新三板”上市的企业。科技创新持续增强，市政府与武汉大学正式签订科技合作协议，全市高新技术企业达到50家，列全省第二；高新技术产业增加值90亿元，占规模以上工业增加值的18%；新组建省级工程技术研究中心2家、省级优势创新团队2个，专利申请量达1000件，我市荣获省政府质量奖考核第一名。

　　与此同时，政府自身建设继续加强。深化行政审批制度改革，市级行政审批服务事项审批时限在原来压缩47%的基础上再压缩30%以上，审批环节减少20%以上，列全省第一，丰城被列为“全国政务公开和政务服务试点市”，市公安局被列为“全国公安机关执法示范单位”，市行政服务中心公安窗口荣获“全国优秀公安基层单位”，市交通战备办被国家国动委评为“十一五”以来先进单位。坚持向人大报告工作、向政协通报情况，认真接受人大法律监督和政协民主监督，166件人大代表建议、270件政协委员提案全部办复。政府信息公开领域进一步拓展，行政执法更加规范，规范性文件报备率、行政复议率、投诉回复率达到100%，市人防办荣获“全国人防信息化建设先进单位”，铜鼓获得“全国法治县创建活动先进单位”。扎实开展“集中整治影响发展环境的干部作风突出问题”活动，我市在全省会议上作了典型发言。落实政府系统党风廉政建设责任制。审计监督及审后整改扎实推进。国防后备力量建设、国家安全、外事侨务、民族宗教、涉台事务、邮政通信、烟草、盐业、气象水文、防震减灾、无线电管理、妇女儿童、统计、档案、扶贫、老龄、残疾人和红十字会等工作都取得了新成绩。

　　各位代表，过去的一年，我们牢牢把握“稳中快进、难中取胜”的总基调，咬准目标不放松，勤爬坡，真流汗，经济社会发展全面推进，取得了预期的成绩。这得益于省委、省政府和市委的坚强领导，离不开市人大、市政协的监督支持，凝聚了全市人民的勤劳智慧。在此，我代表市人民政府，向付出辛勤劳动的全市人民，向给予政府工作大力支持的人大代表和政协委员，向各民主党派、工商联、无党派、人民团体、驻宜部队、民兵预备役、武警官兵、公安干警和社会各界人士，向所有关心支持宜春发展的同志们、朋友们，表示崇高的敬意和衷心的感谢！

　　在肯定成绩的同时，我们也清醒地认识到发展中的困难和问题：经济总量不大、产业结构不尽合理、重大骨干项目不多，赶超发展的基础还不牢固；农民持续增收难度较大，部分群众生活困难；社会不稳定因素仍然存在，维护安全稳定任务艰巨；政府机关作风和干部干事创业精神与新形势、新任务的要求还有差距，突出表现在敢于担当意识不强、破解难题办法不多、工作落实力度不够，等等。对此，我们将高度重视，采取有力措施加以解决，努力把各项工作做得更好。

二、2013年目标要求

　　2013年是全面深入贯彻党的十八大精神的开局之年，是实施“十二五”规划承前启后的关键一年，做好全年各项工作，意义重大。新的一年，挑战依然严峻，机遇不容错过。一方面，欧债危机仍未结束，世界经济复苏的不确定性因素增多，国内经济下行压力增大，稳增长任务十分艰巨。营业税改征增值税试点的逐步推开，结构性减税影响加深。同时政策性支出增多，仅市本级增支就达3亿元以上，财政收支平衡压力大。此外，随着政府债务进入偿还密集期，偿债压力加大，既要防止出现支付风险，又要严格控制债务规模。另一方面，中央经济工作会议强调，今年继续实施积极财政政策和稳健货币政策，牢牢把握扩大内需这一战略基点；鄱阳湖生态经济区战略的深入实施，特别是随着明月山机场顺利竣工，杭长铁路客运专线抓紧推进，我市即将迎来航空、高铁时代，发展条件更为优越。我们要将宜春放在全省科学发展、进位赶超、绿色崛起的目标中去谋划，放在全面建成小康社会、努力建设美丽中国的大局中去考量，在迎接挑战中抢抓机遇，在战胜困难中赢得主动，“登高”提速，“临空”起航，奋力开创宜春科学发展的新时代！

　　根据中央、全省的部署和要求，结合宜春实际，今年政府工作的总体要求是：全面贯彻落实党的十八大精神，以邓小平理论、“三个代表”重要思想、科学发展观为指导，紧紧围绕主题主线，抓住并用好机场和高铁即将开通的机遇，继续坚持“稳中快进、难中取胜”的总基调，继续着力推进新型工业化、信息化、城镇化和农业农村现代化，突出抓好重大项目和重点产业建设，突出抓好服务业发展，突出抓好改善民生和创新社会管理，确保主要经济指标继续保持赶超进位的好势头，确保社会安全稳定，确保幸福宜春建设取得新成效。

　　全市经济社会发展主要预期目标是：生产总值增长11%；财政总收入和地方财政收入均增长18%；规模以上工业增加值增长15%；全社会固定资产投资增长25%；社会消费品零售总额增长13%；实际利用外资和外贸出口均增长10%；城镇居民可支配收入和农村居民可支配收入均增长13%；城镇化率提高2个百分点；城镇登记失业率控制在4.5%以内，人口自然增长率控制在8‰以内，居民消费价格指数控制在4%以内。

三、2013年工作重点

　　目标既定，任务已明，关键在落实，核心在操作。我们将全力以赴，继续深入实施“十大幸福工程”，打好“产业升级三年强攻战、城市建设三年升级战、农业产业化三年扩张战、服务业三年大会战”四大战役，培育项目气场，打造城市气势，增添文化气派，弘扬宜春气度。

　　（一）坚持项目带动，增强发展后劲

　　紧扣项目建设这一核心不动摇，进一步整合资源，聚力推进，实现重大项目新突破。

　　捕捉信息。调动一切因素，挖掘一切潜力，聘请一批专职信息员和招商顾问，继续开展重大项目信息征集工作，理出一批高质量的重大项目线索，确保全市新掌握亿元以上项目线索不少于300条，其中10亿元以上重大项目线索不少于50条。

　　精细包装。密切关注国家投资政策，充分发挥自身优势，不断调整项目库，实现滚动储备和及时充实，做到申报一批、建设一批、投产一批、储备一批，力争全市新增80个重大项目列入省级调度。

　　招大引强。以招大引强、集群承接为主线，既注重招商数量更注重质量，既注重规模更注重效益。全市围绕锂电新能源、机械电子、电力能源、医药食品、化工、建材等重点产业，力争引进亿元以上项目100个，其中10亿元以上重大项目20个，50亿元以上重大项目3-4个，100亿元以上重大项目1-2个。

　　快速落地。强力推进河北御捷、深圳盟立、赣锋锂业、西锐航空、中国瑞林、昆庆纺织、锦华纺织、长新电源、蓝恒达二期、飓风化工、广州王老吉、绿色照明、盛泰昌等一批项目的开工、建设。市级抓好10亿元以上重大项目调度，全市重点抓好100个项目建设。力争全市新开工建设10亿元以上重大项目30个，亿元以上工业项目突破80个。

　　优质服务。完善重大项目协调推进机制，切实做到大项目大支持，快项目快支持，好项目好支持，真项目真支持。健全审批“绿色通道”，实施重大项目代办制，规范收费行为，降低企业成本，着力打造“宜春速度”，一天能办成的事决不用两天，一块钱能办成的事决不花两块钱，一次能办成的事决不分两次办。实行领导干部挂钩服务机制，明确市领导挂钩服务100条重大项目线索推进和100个重大项目落地任务。

　　（二）推进“两化”进程，加速赶超步伐

　　以新型工业化为“发动机”，引领城镇化水平提升；以新型城镇化为“加速器”，推动工业产业升级，实现“两化”良性互动发展。

　　加快工业化，突出“三抓”：一抓优势产业。重点推进锂电、机电、绿色食品和生物医药等主导产业发展。锂电产业在资源掌控、重大项目引进、现有企业做大做强上力求新突破。机电产业以宜春重工、江特电机等为龙头，推进产业配套和协作。绿色食品产业着力做强四特酒公司、春丝食品、旺旺食品等骨干企业，做大“富硒”和“有机”品牌。生物医药产业依托樟树、袁州医药产业基地，抓好行业新版标准改造和新药开发。二抓园区建设。按照“做大总量、彰显特色、完善配套、提升效益”的原则，加快推进产业集群，做大做强一批特色产业园区，力争丰城高新技术产业园突破400亿，宜春经开区、高安、樟树、上高、奉新五个园区均突破300亿。切实加强生态文明建设，巩固六大环保专项整治成果，坚决不引进高污染项目，坚决完成节能减排任务，坚决整治影响发展质量和人民健康的突出污染问题，真正做到“既要金山银山，又保绿水青山”。实施“品牌园区”创建，整治和防范园区污染，实现省级生态工业园区全覆盖，创新中国成立家产业示范基地1-2个，宜春经开区争取晋升国家级。三抓龙头企业。促进生产要素向龙头企业集中，加快培育一批“航母型”、“旗舰型”企业。抓好兴发铝业、福斯特新能源等扩建续建，加强跟踪服务，扩大生产能力。力争全市新增规模以上工业企业80户，主营业务收入超10亿元以上企业达到25户，其中济民可信、仁和药业、四特酒公司均突破50亿元，年纳税百强企业标准提高到1500万元。继续支持中央、省属企业做大做强。面对激烈竞争，引导企业改变过去单打独斗的发展方式，学会抱团取暖，提升竞争能力。

　　推进城镇化，做到“三注重”：一是注重规划引领。深化城市总体规划和控制性详规，加强环卫设施、户外广告等专业规划编制，突出地域性、彰显高品味、体现多样化，杜绝建设牵着规划走。合理组织村镇建设项目用地与布局，完成省级试点村规划编制。二是注重功能配套。按照“理性改造老城区，加快建设新城区，规划先行谋长远，严格管理上水平，功能配套利民生”的原则，推进中心城区科技之窗、北湖公园、宜阳森林公园、宜阳南路跨铁路立交桥建设，启动渥江湿地公园、动物园、植物园建设。完善城市给排水和污水管网工程，启动中心城备用水源、污泥处理建设。加快垃圾中转站、公厕新建和改造，推进生活垃圾分类收集与处理，探索环卫市场化运营机制。大力发展公共交通，落实公交优先政策。完善道路交通设施，加快公共停车场建设，改善市民出行条件。开展“中国人居环境奖”创建，启动“数字城管”建设，提高城市管理水平。三是注重城乡协调。各地围绕“三年拉框架，再造新城区”目标，加大投入，完善功能，提升城市品位。争取南昌-万载-上栗高速公路、宜丰-上高-新余一级公路、昌吉赣城际铁路开工，做好岳吉铁路前期工作。坚持集约节约用地，提高供地率和使用率。加快省级示范镇和市级重点镇建设，强化村镇规划，提升集镇管理水平，拓展新型城镇化发展空间。放宽户籍限制，促进在城镇有稳定居所、稳定职业、稳定经济来源的农民有序转变为城镇居民。在推进城市化中，要体现政府意志，引领城市整体战略；发挥资本意图，增强城市发展动力；征询民众意愿，表达真实生活需求；倾听专家意见，完成优质城市规划，努力使宜春成为人人向往的繁荣之地、创业之城、幸福之家。

　　（三）增创农业优势，建设秀美乡村

　　加大统筹力度，加快现代农业和新农村建设，推动城乡一体化发展，促进农民增收、农业增效、农村增色，实施“四大工程”。

　　一是水利强农工程。加大农田水利基础建设，抓好病险水库除险加固，推进丰东、锦北、袁北等大型灌区续建配套与节水改造。做好四方井水库、奉新鹅婆岭水库前期工作。加快高标准农田、中小河流治理、水土保持、农村饮水安全等项目建设。开展坡耕地水土流失综合治理试点。

　　二是产业兴农工程。严格耕地保护，确保粮食播面稳定在900万亩以上，粮食总产稳中有升。大力培育新型农业经营主体，促进农业规模化、集约化、标准化发展。加快推进粮食、畜禽、油茶、毛竹、花卉苗木、有机富硒农产品等六大农业重点产业发展，抓好蔬菜、茶叶、果业标准园和80个现代农业示范点建设，力争新增国家级现代农业示范区1个、省级现代农业示范区2个。

　　三是绿色富农工程。加大政策扶助力度，新造高产油茶林11万亩、花卉苗木基地10万亩、毛竹丰产林基地16万亩。稳步实施造林绿化“一大四小”工程，推进“国家森林城市”创建，积极开展“森林城乡、绿色通道”建设，突出抓好高速公路、国省道和铁路两侧绿化带建设，努力建成通道绿化景观林带和绿化精品。

　　四是生态惠农工程。以“让村庄美丽、让农民幸福”为目标，大力开展“和谐秀美乡村”创建。加快新农村建设和扶贫开发，抓好高速公路和高铁沿线村庄综合整治，深化村镇联动、村落连片整治。继续实施农村清洁工程，推广靖安城乡一体化垃圾处理模式，逐步实现农村垃圾处理减量化、资源化、无害化，全面改善农村生产生活条件。

　　（四）加强扶持引导，壮大提升服务业

　　抓紧制定高铁、机场片区规划，积极谋划航空经济和高铁经济板块产业发展，着力将宜春打造成“赣湘边界的服务业高地”，力争2013年全市服务业增加值占GDP比重达26%以上，服务业总投资占全社会投资比重达24%以上，服务业地税收入占全部地税收入比重达69%以上。

　　做大做强旅游产业。围绕打造“全国知名养生休闲度假胜地”的总体定位，突出“月亮之都、禅宗圣地、温泉之乡”的地方特色，继续打好旅游升级战，推进明月山创新中国成立家5A级旅游景区，宜春迎宾馆、丰城花园酒店创评五星级旅游饭店，办好第七届月亮文化节。加快宜春禅博园、明月山缆车改造和山顶观光小火车、仰山国际温泉禅修中心，樟树养生天堂旅游综合开发，丰城养生硒谷富硒温泉，靖安生态经济示范基地、恒茂金罗湾温泉度假村、武侠世界文化产业园，上高九峰景区，铜鼓天柱峰景区、汤里温泉和福润温泉等重大旅游项目建设，形成核心竞争力。深化赣西三市一县旅游合作，拓展新兴市场，力争全年接待游客量突破2000万人次，旅游综合收入达到150亿元。

　　大力发展新型服务业态。重点发展现代物流、电子商务、科技信息等新兴服务业，推动服务业与信息化、工业化深度融合。优化提升商贸流通业，做好大型商贸综合体的规划布局，争取中心城区时代广场、红星美凯龙等商业综合体年内开工，推进丰城林安国际商贸城、宜丰红商城等在建商业综合体建设，加快再生资源回收利用、汽车大市场等项目建设，完善社区便民消费网络，形成多元化、多层次的商业服务网络。大力培育现代物流业，加快赣西物流中心、宜春铁路货场、宜春经开区服务业基地、高安生产性服务业基地、樟树港铁口岸作业区的建设和改造提升，引进培育物流龙头企业，建立物流联动机制，重点发展产业基地、专业市场、城乡配送等物流产业。加快发展节庆、商务会展、体育健身休闲等文化产业，培育一批具有较强竞争力的文化企业。大力引进一批专业从事电子商务研发、销售、服务的大企业、大集团，扶持发展一批网商企业，积极培育一批为电子商务服务的物流配送、第三方支付和中介服务企业，加快形成以行业网站、电子商务应用为重点的新格局。

　　繁荣发展金融业。加强银企对接，降低企业融资成本，力争新增贷款130亿元、新设小额贷款公司3家。积极引导和扩大社会投资，探索民间融资规范化和阳光化途径。力争新引进外埠银行2家，逐步把宜春农商行打造成有影响力的地方银行。抓好直接融资，争取1家企业成功上市、2家企业通过辅导验收并上报国家证监会。

　　（五）狠抓改革创新，激发内生活力

　　随着改革进入攻坚期和深水区，我国的“人口红利”逐渐消减，改革本身势必挑起科学发展的大梁，形成新的“改革红利”。可以说不改革不发展、小改革小发展、大改革大发展。

　　深化重点领域改革。做好企业改革扫尾工作。推进财税体制改革，加强财政预算绩效管理。抓好丰城省级县域经济综合配套改革试点。加快医药卫生体制改革，巩固实施基本药物制度，推进县级公立医院综合改革试点。继续深化文化体制改革，进一步激发转企改制后的国有文艺院团活力。抓好基层农技推广服务体系改革与建设。

　　强化创新驱动。加大与国内外高校、科研院所的深度合作，力争实施200个科技攻关或科技成果转化项目。推进锂电新能源高新技术孵化园建设，争取宜春锂电新能源高新技术创业服务中心建成省级创业服务中心，丰城中国生态硒谷农业科技园升格为国家级农业科技园，全市高新技术产业增加值突破120亿元。实施名牌带动战略，大力推进质量兴市，力争新增国家地理标志保护产品2个，驰名商标2个，国家级标准化技术委员会1个。加强人才队伍建设，抓好企业和特殊行业急需人才的引进、培养和使用，强化农村实用人才培养。

　　扩大对外开放。拓展产业招商领域，加强与央企、省属国企的合作对接，力争全年引进省外资金300亿元以上；实际利用外资突破5亿美元。完善出口促进政策，加快集装箱场站和质量安全示范区建设，推动出口主体和出口市场多元化，争取外贸出口总额突破15亿美元。加大对小微企业的政策扶持，推进全民创业。

　　（六）切实改善民生，促进社会和谐

　　多办广受百姓期盼的实事，多做惠及百姓生活的好事，使百姓持续得实惠，生活不断有改善。

　　以人为本惠民生。以全民覆盖为目标，认真落实更加积极的就业政策，统筹推进城乡社会保障体系建设。进一步完善社会救助体系，着力提高城乡低保水平，开展城镇居民医保、大病医疗救助和自然灾害救助工作，健全农村留守儿童帮扶机制。加大保障性住房建设、农村危房、城中村改造力度，切实保障困难群众和弱势群体生产生活，确保城乡基本医疗保险参保率稳定在95%以上，城镇居民医疗保险补偿率稳定在70%以上。加强商品房工程质量监管，规范小区物业管理，提高服务水平。继续开展各级干部进农村、进社区、进企业帮扶活动，努力为基层群众和企业解决实际问题。

　　统筹发展促和谐。坚持优先发展教育事业，加快学校项目建设，有效化解城区教育 “大班额”问题。推进义务教育学校标准化建设。启动县级教育专项规划编制工作。加强文化惠民服务，抓好市博物馆、图书馆、档案馆等开发利用。创新长效管理机制，巩固和发展国家卫生城市成果。加快市、县、乡、村四级医疗机构网络建设，加强医德医风建设，切实解决群众“看病难、看病贵”问题。推进全民健身工作，办好市第四届运动会。加快数字影院建设，实现县县有数字影院。继续抓好重大疾病免费救治，适时推广实施“爱童工程”。推进戒毒康复中心建设。完善计划生育利益导向机制，提高出生人口素质。

　　创新管理保稳定。落实企业安全生产责任制，加大监管力度，强化隐患排查，预防和减少较大以上安全事故的发生。健全社会矛盾大排查大调处工作体系，完善信访制度，进一步落实重大决策重大项目社会稳定风险评估机制。加强食品药品监督管理，全力创建食品药品安全示范市。抓好重大动物疫病防控，加强粮油、蔬菜质量监测。扎实开展“六五”普法。健全突发事件预警和应急处置机制，加快搜救犬基地和应急物资储备基地配套建设。抓好国防动员、民兵预备役、双拥模范城创建、军民融合、人民防空等工作，加强国防后备力量建设。着力构建社会管理“三网”工作体系，夯实基层基础。深化平安建设，完善立体化社会治安防控体系，加强对流动人口和特殊人群的服务管理，依法防范和惩治违法犯罪，不断提升公众安全感和满意度。

　　各位代表，在去年《政府工作报告》中，我们向全市人民郑重承诺，在努力完成各项目标任务的同时，突出抓好20个重大民生项目。一年来，市政府坚持十天一调度，每月一通报，不定期现场督查，各地、各有关部门凝心聚力、齐抓共管，确保了项目顺利推进。135个子项目中，除新建袁州教育园区普通高中、宜春大桥改造、环北湖路建设经市委、市政府研究决定暂缓实施外，有119个达到或超过进度要求；计划年内完工的96个项目中，91个顺利完工，完工率达94.8%。在推进去年有关项目续建的基础上，我们将继续按照“项目化、时间表、责任人”的要求，重点抓好以下“双十”工作。

　　（一）十大基础工程

　　1. 启动宜春中心城湖田片区连片开发。

　　2. 推进宜春中心城区环城南路建设。

　　3. 争取宜春新火车站综合交通枢纽竣工并投入使用。

　　4．完成明月山机场建设，确保上半年通航。

　　5．推进万宜高速公路建设。

　　6．推进杭长铁路客运专线建设。

　　7．抓紧昌樟高速公路拓宽改造。

　　8．抓好靖安洪屏抽水蓄能电站续建。

　　9．推进丰厚一级公路建设。

　　10．抓好里睦等233座中小型病险水库除险加固。

　　（二）十大民生实事

　　1．推进保障性住房建设。力争全市建设保障性安居住房35417套，其中中心城区完成廉租住房712套、公共租赁住房3016套、城市棚改9110套。

　　2．继续化解城区“大班额”问题。抓好袁州学校等14所学校新建，推进宜春七小等3所学校改扩建。

　　3．新建一批综合性医院。推进宜春市人民医院（北院）和宜春市儿童医院建设，启动丰城市人民医院住院大楼建设。

　　4．加大就业创业扶持。新增城镇就业4.7万人、失业人员再就业2.2万人，“零就业家庭”实现100%动态就业援助，新增农村劳动力转移就业6.6万人。

　　5．提升社会保障水平。提高城市和农村低保标准和补差水平，力争城市低保标准达到400元，补差水平标准达到240元；农村低保标准达到200元，补差水平达到120元。稳定新型农村养老保险参保人数 158万人、城镇居民医疗保险参保人数145万人；新增养老保险参保人数2万人。

　　6．改善农村交通条件。争取全年完成县乡道升级、农村客运网络公路改造、新修通村民小组水泥（油）路700公里，行政村通客车率达到92%。新建“六位一体”功能的农村公路综合服务站6个、农村客运站3个、农村候车亭178个。

　　7．完善环保配套设施。加快宜春经开区和丰城新城区等污水处理厂管网建设；推进高安建筑陶瓷产业基地、上高工业园等4个园区污水处理厂建设；加快地表水和饮用水水源水质自动监测站建设，完成袁州西村镇张坊村自动监测站等3个项目建设。

　　8．实施便民服务工程。全市所有乡镇（街道）规范建设社会管理服务中心。抓好“菜篮子”、“米袋子”建设，启动农贸市场升级改造，中心城区完成10个农贸市场标准化升级改造(新建），新建9个运动游园，试点新设2个市民幸福书屋。

　　9．整治中心城区交通拥堵。加快智能交通指挥系统、平安智能街区（社区）建设，推进明月大桥、明月北路延伸等项目建设，抓好小街小巷及宜阳大道、中山路等道路油化改造。

　　10．抓好防震减灾工作。推进县市防震减灾“九个一”工程建设。

　　各位代表，建设幸福宜春的时代进程，既宏伟壮丽，又充满艰辛。但是，任何困难都不能改变宜春人民创造幸福生活的强烈愿望，任何挑战都不能动摇宜春人民进位赶超的坚定信心，任何障碍都不能阻挡宜春人民绿色崛起的豪迈步伐。我们将牢记习总书记“敏于行、慎于言，降虚火、求实效，实一点、再实一点”的教导，围绕“职能科学、结构优化、廉洁高效、人民满意”的服务型政府的总体目标，切实加强政府自身建设，让政府成为人民雨中的大伞、路上的驿站、心里的靠山。我们将强化进取意识。坚持“重点论”和“两点论”相统一，把精力集中在想干事上，把本领体现在会干事上，把目标锁定在干成事上，注重操作，敢于担当，减少负效应，聚合正能量。强化为民意识。坚持人民利益至上，心里装着群众、感情贴近群众、工作依靠群众，作决策充分尊重民意，办实事充分体现民需，抓改革充分保障民利，切实解决好群众最急、最盼的问题。强化法制意识。自觉接受人大法律监督和政协民主监督，加强审计监督、行政监察，全面推进政务公开，健全农村集体“三资”管理，确保行政权力依法、公开、公正运行。强化清廉意识。职务是为民谋利的平台，岗位是为民奉献的战场。全体公务人员要严守《廉政准则》，自觉抵制和克服浮躁之风、庸俗之风、奢靡之风，做到知恩不惑、知足不辱、知止不殆，在干事创业中实现人生最大价值。

　　各位代表，深入学习贯彻党的十八大精神，在市委的坚强领导下，政府工作如果概括为一个字就是“干”，两个字就是“落实”，三个字就是“执行力”。站在新的历史起点上，面对全市人民的期盼，我们深知宜春的发展慢不得，必须加快；我们深感肩上的担子沉甸甸，不敢懈怠。我们将紧紧依靠全市人民，走好每一步，跨越每道关，为建设幸福宜春而努力奋斗！
